# Unveiling the Unique Reinforcement Mechanisms of Coiled Carbon Nanotubes and Their Hydrogenated Counterparts in Asphalt Modification

**DOI:** 10.3390/nano15231805

**Published:** 2025-11-29

**Authors:** Fenghua Nie, Hang Lin, Xing Su, Ke Ou, Yifan Chen

**Affiliations:** 1 School of Resources and Safety Engineering, Central South University, Changsha 410083, China; 2 China Railway No.5 Engineering Group Co., Ltd., Guiyang 550000, China

**Keywords:** hydrogenated coiled carbon nanotubes, asphalt, nanocomposites, molecular dynamics simulations, pullout performance

## Abstract

Coiled carbon nanotubes (CCNTs) and hydrogenated coiled carbon nanotubes (HCCNTs) are advanced nanomaterials recognized as having exceptional mechanical properties and the potential to enhance composite materials. This study examines the pullout performance of CCNTs and HCCNTs in reinforcing asphalt nanocomposites through molecular dynamics simulations. Results indicate that CCNTs generally exhibit superior maximum pullout forces compared to HCCNTs, particularly at lower temperatures, suggesting stronger adhesion within the asphalt matrix. Conversely, HCCNTs demonstrate enhanced stability and flexibility under varying temperature conditions, allowing them to maintain more pullout energy over longer distances. Molecular movement analyses reveal that CCNTs facilitate greater displacement of asphalt molecules, indicating stronger interactions, while spatial configuration studies show that CCNTs attract more asphalt molecules in close proximity, enhancing their effectiveness. Additionally, HCCNTs possess a larger free volume, which may increase the risk of nano-damage but also allows for greater flexibility during the pullout process. This research highlights the distinct roles of CCNTs and HCCNTs in improving asphalt’s mechanical properties, ultimately contributing to the development of more resilient and sustainable asphalt pavements.

## 1. Introduction

Asphalt is an essential material widely utilized in road engineering and construction. In road engineering, it serves as a primary component for paving highways, streets, and airports, thanks to its excellent mechanical properties and capacity to endure heavy traffic loads. Its durability and flexibility make it ideal for creating smooth, long-lasting surfaces capable of withstanding various weather conditions [1]. However, asphalt faces challenges such as susceptibility to cracking, rutting, and aging, especially under extreme temperatures and heavy traffic [2,3]. These issues can result in significant maintenance costs and reduced service life. In recent years, modified asphalt has gained considerable attention due to its enhanced performance characteristics compared to traditional asphalt [4]. One of the primary benefits of modified asphalt is its improved durability, which enables it to better withstand harsh environmental conditions and prolonged exposure to traffic stresses. This increased durability ultimately leads to a longer service life and lower maintenance costs, making modified asphalt a more economical choice in the long run.

Agglomeration of CNTs can lead to localized stress concentrations, which can negatively impact the mechanical properties of the composites, such as tensile strength and toughness. Furthermore, the orientation of the nanotubes during the mixing process can significantly influence load transfer efficiency and overall composite performance [5]. Recent studies on functionalized CNTs have shown significant advancements in enhancing the mechanical properties, durability, and compatibility of asphalt and polymer composites [6,7]. The functionalization of CNTs—through the introduction of carboxyl, hydroxyl, or amino groups, or by using surfactants via non-covalent methods—greatly enhances their dispersion in polymer and asphalt matrices. This uniform dispersion reduces agglomeration, resulting in improved interfacial bonding, enhanced fracture resistance, increased fatigue life, and better thermal stability in both asphalt composites and polymer nanocomposites [8,9]. In asphalt materials, functionalized CNTs help mitigate the negative effects of moisture and chemical runoff, leading to modified mixtures with improved fracture toughness and energy absorption under intermediate conditions. Mechanistically, hydrogenation differs from conventional functionalization, which primarily introduces polar or reactive groups to enhance affinity with the matrix. Instead, hydrogenation fundamentally alters carbon hybridization and surface energy, affecting dispersion, wettability, and compatibility with various matrices. While both functionalization and hydrogenation help reduce CNT agglomeration and improve integration with the matrix, hydrogenated CNTs may offer enhanced hydrophobicity and better compatibility with hydrocarbon-rich matrices like asphalt [10,11].

Coiled carbon nanotubes (CCNTs) are a novel form of carbon nanotubes distinguished by their unique spiral structure, which sets them apart from traditional carbon nanotubes (CNTs) [12,13]. This distinctive configuration not only contributes to their exceptional mechanical properties but also enhances their electrical conductivity and thermal stability. The spiral shape of CCNTs increases their strength and rigidity, making them particularly suitable for a wide range of applications, including advanced composites and nanotechnology. Compared to traditional CNTs, CCNTs offer several advantages, such as improved flexibility and a larger surface area for interaction with matrix materials, leading to enhanced performance in composite materials [14]. Research indicates that CCNTs can significantly enhance the thermomechanical properties of carbon fiber-reinforced polymer composites through improved interfacial bonding and mechanical interlocking [15]. This coiled structure can improve both the fracture toughness and mechanical strength of the composites, even in the absence of direct chemical bonding between the nanotubes and the matrix [16]. Various geometrical aspects of CCNTs, including inner diameters, helix angles, and tube diameters, are examined in pullout tests of polymer composites, revealing that the maximum tensile force decreases with larger inner diameters, higher helix angles, and smaller nanotube diameters [17]. When incorporated into asphalt, CCNTs significantly enhance its thermomechanical properties, leading to improved resistance to deformation and a reduced risk of rutting and cracking in pavement structures [18].

However, incorporating CCNTs into composites poses challenges, including increased brittleness and potential dispersion issues [16], which can adversely affect the overall performance of the material. To address these challenges, hydrogenation is applied to enhance the compatibility of CCNTs, improve their mechanical properties, and mitigate negative effects in the modified nanocomposites. Studies have demonstrated that the mechanical properties of CCNTs with varying geometries improve through hydrogenation, with findings indicating that increasing hydrogenation beyond 30% enhances their stretchability, while temperature has a minimal impact [19]. The resulting hydrogenated coiled carbon nanotubes (HCCNTs) show improved compatibility with matrix materials, which can further enhance the mechanical performance of the composite. However, it is still unclear whether this enhanced compatibility of HCCNTs leads to improved mechanical properties in asphalt, especially under varying temperature conditions. Consequently, this study aims to investigate the differences in pullout characteristics between CCNTs and HCCNTs in modified asphalt. Importantly, to the best of our knowledge, few studies have examined the application of HCCNTs in modified asphalt nanocomposites, highlighting a significant gap in the literature that necessitates further investigation.

Molecular dynamics (MD) simulation is a powerful computational technique that models the behavior of atoms and molecules over time, based on the principles of classical mechanics [20,21,22,23,24,25]. This method allows researchers to investigate the interactions and movements of particles at the atomic level, making it particularly valuable in materials science. One of the key advantages of MD simulations is their ability to provide detailed insights into molecular interactions, enabling the exploration of complex systems that would be difficult to analyze experimentally [26,27]. In the study of asphalt and its modified materials, MD simulations play a crucial role in enhancing our understanding of molecular interactions and performance characteristics. They allow researchers to observe how different components of asphalt interact at the nanoscale, leading to improved formulations [28]. Even though MD simulations performed at the nanosecond scale may not fully capture the slow relaxation processes or viscoelastic behavior of asphalt under realistic loading rates, they are valuable for providing insights into the instantaneous interactions occurring at the moment of asphalt material interaction. Additionally, MD simulations can predict the mechanical properties and durability of asphalt mixtures under various environmental conditions, facilitating the design of novel paving materials [29]. By optimizing formulations based on these insights, MD simulations contribute significantly to the development of asphalt-based materials that are not only more efficient but also more sustainable, ultimately leading to longer-lasting and more effective paving solutions.

The objective of this study is to investigate the pullout performance of CCNTs and HCCNTs in reinforcing asphalt. Six different CCNTs and HCCNTs geometries are proposed and compared, and the reinforcing mechanisms are revealed through MD simulations. Results indicate that CCNTs generally exhibit superior maximal pullout forces compared to HCCNTs, especially at lower temperatures, which suggests a stronger adhesion within the asphalt matrix. In contrast, HCCNTs demonstrate enhanced stability and flexibility under varying temperature conditions, enabling them to maintain more pullout energy over longer distances. Analyses of molecular movement show that CCNTs facilitate greater displacement of asphalt molecules, indicating stronger interactions, while spatial configuration analyses reveal that CCNTs attract more asphalt molecules in close proximity, thereby enhancing their effectiveness. Overall, this study highlights the unique characteristics and roles of CCNTs and HCCNTs in enhancing the mechanical properties of asphalt, which paves the way for the advancement of more durable and sustainable asphalt pavements.

## 2. Model and Methodology

### 2.1. Atomistic Model

**Figure 1:**
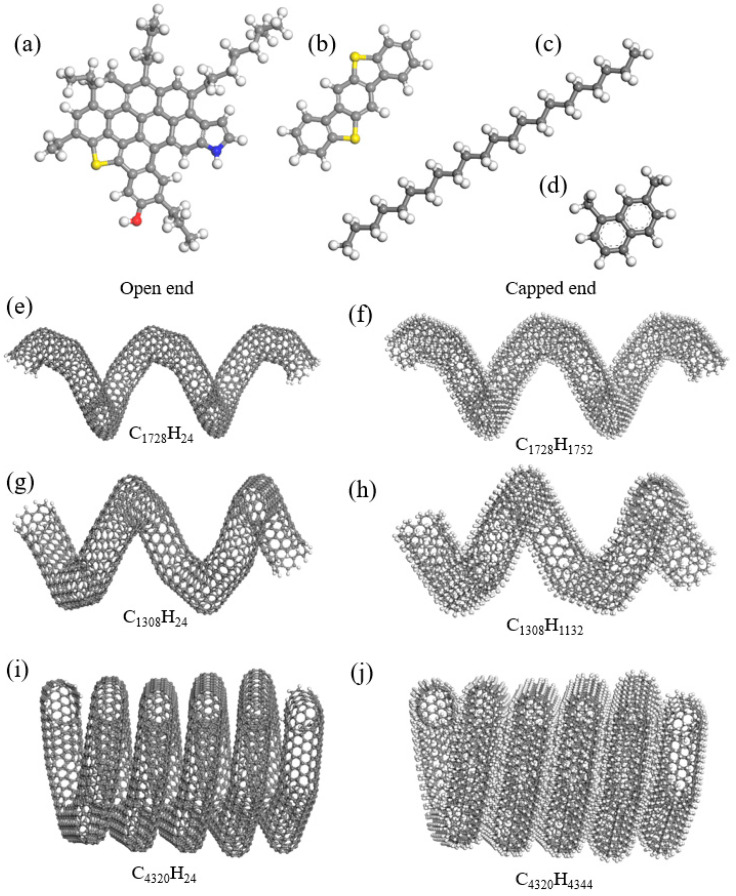
Molecular structures of (**a**) asphaltene, (**b**) polar aromatics, (**c**) saturate, (**d**) naphthene aromatics, and (**e**) CCNT1, (**f**) HCCNT1, (**g**) CCNT2, (**h**) HCCNT2, (**i**) CCNT3 and (**j**) HCCNT3 (The carbon, oxygen, nitrogen, sulfur, and hydrogen atoms are presented in gray, red, blue, yellow, and white colors, respectively).

The four-molecule asphalt model is selected for this study, as it has been widely used in molecular dynamics (MD) simulations of asphalt [30]. This model includes asphaltene, polar aromatic, naphthene aromatic, and saturate molecules. Asphaltene molecules are relatively large and heavy, with a carbon-to-hydrogen ratio of 1:1.2, a mean atomic mass of approximately 750 g/mol, and a diameter ranging from 10 to 20 Å [31]. Based on these characteristics, a modified asphaltene molecule is chosen, as illustrated in Figure 1a. Benzobisbenzothiophene is selected as the polar aromatic molecule according to geochemical literature [32]. Considering polarity and elemental fractions, 1,7-Dimethylnaphthalene is chosen as the representative naphthene aromatic molecule. N-docosane is selected as the saturate molecule due to its similar melting and boiling points. After defining these molecules, representative molecules of individual asphalt components are packed to match the elemental mass fraction, atom ratio, and aromatic/aliphatic ratio observed in real asphalt from experimental data. The mass ratios of the different asphalt molecules are listed in Table 1, aligning with the physical and chemical properties found in experimental results [33]. The four-component model may not accurately predict the physical and chemical characteristics of various asphalt mixtures, limiting its applicability across different asphalt sources. However, its simplified structure allows for faster computational processes, making it feasible to explore a wide range of conditions and scenarios without excessive resource demands. Three typical CCNT structures, namely CCNT1, CCNT2, and CCNT3, are introduced in this study and depicted in Figure 1b, Figure 1d, and Figure 1f, respectively. These configurations are chosen because they represent the most representative and commonly observed and employed CCNT structures in the current literature [34,35]. The selected structures are based on their representative characteristics that align with the properties we aimed to investigate, particularly in terms of the mechanical performance and compatibility with asphalt matrices [14]. Specifically, these geometries are chosen due to their favorable chirality and pitch, which are known to influence dispersion and interaction with the matrix. Additionally, their practical applicability in real-world scenarios, where such structures have shown promising results in previous studies [16,36]. To create the desired geometric shapes of CCNTs, heptagon and pentagon defects are introduced at various hexagonal positions within the carbon nanotubes. The construction details of the CCNT molecular structures have been elucidated in our previous study [18]. Additionally, three HCCNTs, namely HCCNT1, HCCNT2, and HCCNT3, are shown in Figure 1c, Figure 1e, and Figure 1g, respectively. The HCCNTs are formed by converting sp^2^ bonds to sp^3^ bonds, which occurs when hydrogen atoms are added to the carbon atoms in the nanotube structure, resulting in a saturated carbon framework and altered electronic properties [19]. All the nanotubes employed in this study are single-walled nanotubes. The CCNT structures are initially placed at the center of the simulation box. The simulation box sizes of the CCNTs/HCCNTs modified asphalt nanocomposites are 60 Å × 60 Å × 60 Å. The atom numbers of CCNT1, CCNT2, CCNT3, HCCNT1, HCCNT2, HCCNT3 modified asphalt nanocomposites are 23,249, 22,940, 23,246, 24,719, 23,879, and 26,841, respectively. Subsequently, the CCNT-modified asphalt nanocomposites are created by packing asphalt molecules into the box using the packing module of Materials Studio 2020.

### 2.2. Forcefield

The Consistent Valence Force Field (CVFF) is an empirical force field specifically designed to accurately model the energetic and structural properties of various molecular systems, particularly organic compounds and polymers [37]. It has been widely applied in studies involving sp^2^/sp^3^ carbon nanostructures interacting with large hydrocarbon mixtures, including CNTs and asphalt [30,38]. It is based on valence bond theory and features a consistent set of parameters that describe bond stretching, angle bending, torsional interactions, and non-bonded interactions. This makes CVFF particularly suitable for simulating complex molecular structures like those found in asphalt, which is primarily composed of hydrocarbons [39,40]. Additionally, CVFF effectively handles the interactions between polymers and nanomaterials, allowing for precise modeling of CCNTs when integrated into the asphalt matrix. The detailed interaction modeling provided by CVFF is crucial for understanding the mechanical and thermal properties of asphalt composites containing CCNTs [41]. Furthermore, the empirical nature of CVFF ensures computational efficiency, enabling researchers to conduct extensive simulations that reveal valuable insights into the molecular interactions and performance characteristics of modified asphalt. The formula of CVFF is presented as follows:

(1) $$\begin{array}{l} E_{total}={\sum_{b}K_{b}(b-b_{0})}^{2}+\sum_{\theta }K_{\theta }{\left(\theta -\theta_{0}\right)}^{2}+\sum_{\phi }K_{\phi }[1+s\cos(n\varphi )]+\sum_{\chi }K_{\chi }[1-\cos(2\chi )]\\ \;\;\;\;\;\;\;\;\;\;+\sum_{nonbond}\left\{\varepsilon_{ij}\left[{\left(\frac{r_{ij}^{o}}{r_{ij}}\right)}^{12}-{\left(\frac{r_{ij}^{o}}{r_{ij}}\right)}^{6}\right]+\frac{q_{i}q_{j}}{\varepsilon_{0}r_{ij}}\right\} \end{array}$$

The energy components in the system consist of both bonded and non-bonded interaction terms. Bonded interactions include bond interactions, bond angle interactions, torsional angle interactions, and out-of-plane angle interactions. In contrast, non-bonded interactions encompass van der Waals and electrostatic interactions. Van der Waals forces are modeled using the Lennard-Jones (LJ-12-6) potential, while electrostatic interactions are represented by the Coulombic function.

### 2.3. Simulation Method

**Figure 2:**
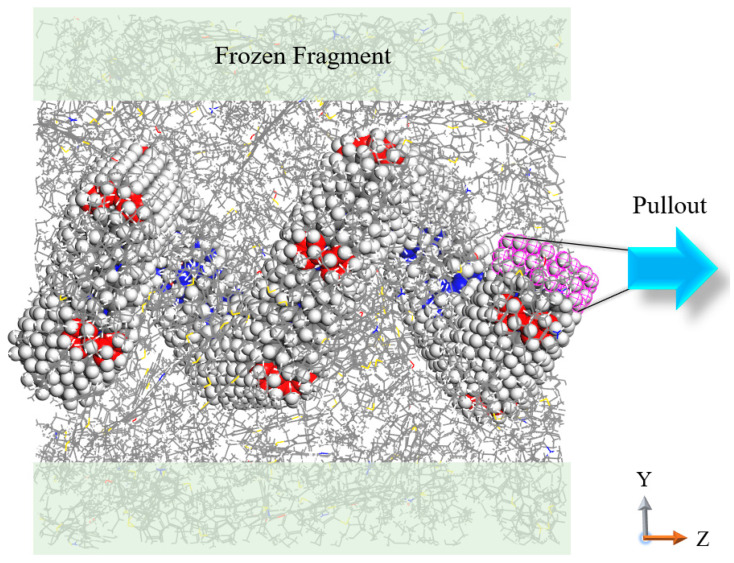
Scheme of HCCNT pulled out from the asphalt matrix (Coordinates axes are defined at the center of mass of the molecules).

The geometry optimization is first conducted on the CCNT/HCCNT modified asphalt nanocomposites using the conjugate gradient algorithm. Following this, the system is first equilibrated for 1 ns under the NPT ensemble, maintaining a constant pressure of 1 atm and a temperature of 300 K to allow for relaxation. To control temperature and pressure, the Nose-Hoover thermostat and barostat are employed, with damping constants set to 100 fs for temperature and 1000 fs for pressure. Following this, an NVT ensemble (constant volume at 300 K) is performed to ensure the thermal stability of the system. For the simulations, all atom pairs are considered within a neighbor cutoff distance equal to their force cutoff plus a skin distance of 2.0 Å. The time step is set to 1 fs, while the cutoff distance for nonbonded interactions and the treatment of long-range electrostatics is established at 12 Å. This choice balances accuracy and efficiency in the simulations. The charge assignments are based on the software defaults in Materials Studio, which are part of the parameters stored within these forcefield definitions. Once the system reaches full relaxation, the pullout test is performed to assess the shear resistance of CCNT/HCCNT modified asphalt nanocomposites, as shown in Figure 2. The simulation box is then expanded by adding a 200 Å vacuum layer along the Z axis, providing sufficient space for the CCNTs and HCCNTs to be extracted from the asphalt matrix. To prevent sliding during the pullout process, the upper and lower boundaries of the asphalt matrix are fixed for 5 Å. The fixed boundaries set at 5 Å can also ensure that the asphalt matrix remains stable and does not pull out along with the CCNT/HCCNT during the simulations. The rightmost sections (around 160 atoms) of the CCNTs/HCCNTs along the pullout direction are held stationary, while the rest atoms can move freely. A constant velocity of 0.001 Å/fs is applied to these atoms to facilitate the extraction of the entire CCNT/HCCNT structures from the asphalt matrix, which is selected based on the established pulling speed value of steered molecular dynamics (SMD) simulations from existing literature [42]. The pullout force and pullout energy values in this paper can be directly obtained through the SMD simulations in the software LAMMPS. The pullout force can be defined by the total force in the pullout direction, while the pullout energy refers to the accumulated PMF (the sum of pulling forces times displacement) in the pullout process. The SMD calculations enable the induction of conformational changes in systems and facilitate the computation of the potential of mean force (PMF) along the designated reaction coordinate using Jarzynski’s equality [43]. SMD simulations have been widely applied in materials science to study the mechanical properties, fracture behaviors, and response of nanomaterials under external forces [44,45]. The displacement quantity of CCNTs/HCCNTs is defined by the displacement of the central point of all the CCNTs/HCCNTs atoms along the pullout direction, which can be calculated by the Z-coordinate after pullout minus the Z-coordinate before pullout. For each case, three parallel simulations are executed individually with independent random seeds, and the results are averaged to minimize random errors. All simulations are performed using the Large-scale Atomic/Molecular Massively Parallel Simulator (LAMMPS 2021) [46].

## 3. Results and Discussion

The density values before and after equilibration are 0.92 g/cm^3^ and 1.04 g/cm^3^ for CCNT1, 0.91 g/cm^3^ and 1.03 g/cm^3^ for CCNT2, 0.98 g/cm^3^ and 1.16 g/cm^3^ for CCNT3, 0.91 g/cm^3^ and 1.02 g/cm^3^ for HCCNT1, 0.90 g/cm^3^ and 1.00 g/cm^3^ for HCCNT2, 0.96 g/cm^3^ and 1.07 g/cm^3^ for HCCNT3, respectively. The obtained density values of CCNT/HCCNT modified asphalt nanocomposites are in accordance with the experimental measurements of CNT modified asphalt nanocomposites, which report values ranging from 1.03 to 1.20 g/cm^3^ [47].

**Figure 3:**
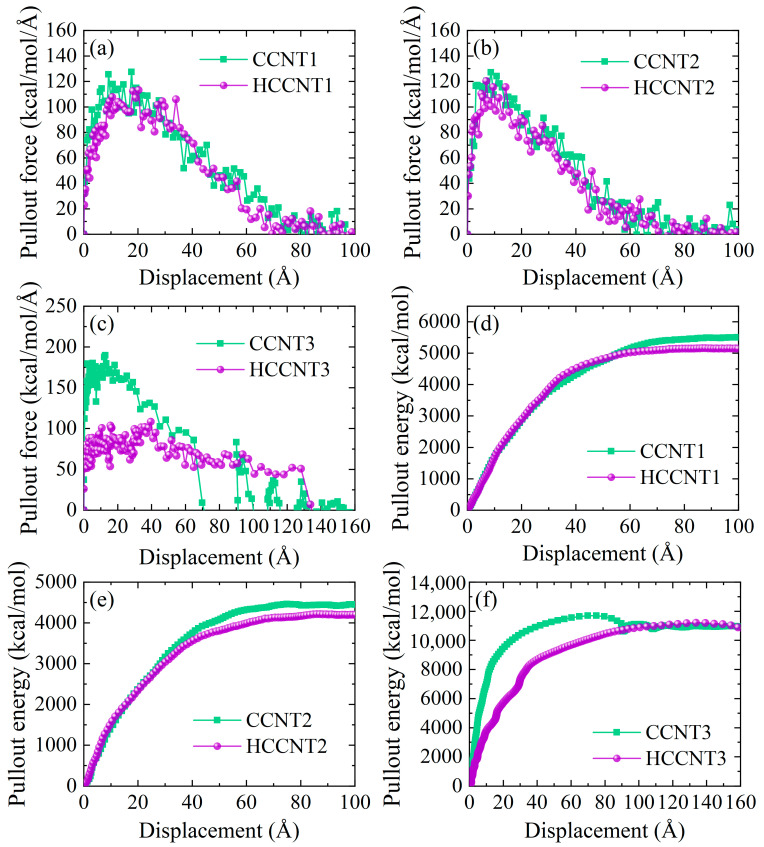
Pullout force of (**a**) CCNT1 and HCCNT1, (**b**) CCNT2 and HCCNT2, (**c**) CCNT3 and HCCNT3. Pullout energy of (**d**) CCNT1 and HCCNT1, (**e**) CCNT2 and HCCNT2, (**f**) CCNT3 and HCCNT3.

The pullout test is crucial for characterizing the mechanical properties of materials, as it provides valuable insights into the adhesion and bonding strength between different components at the microscopic level. The pullout force and pullout energy of CCNTs/HCCNTs during the pullout process are presented in Figure 3. The pullout results obtained in our study fall within a reasonable range and are consistent with existing simulation results reported in the literature [41,48]. It is observed that the pullout force of CCNT1 and CCNT2 increases sharply, reaching the highest value at around 10 Å, before gradually decreasing to zero at approximately 80 Å. Throughout the entire pullout process, the pullout force of CCNT1 and CCNT2 is relatively higher than that of HCCNT1 and HCCNT2. The pullout force of CCNT3 is significantly greater than that of HCCNT3 before reaching 40 Å, with the maximum pullout force of CCNT3 being nearly twice that of HCCNT3 at around 20 Å. After this point, the pullout force of CCNT3 decreases sharply to zero at a distance of 70 Å, while the pullout force of HCCNT3 remains relatively constant at around 50 kcal/mol/Å, ultimately dropping to zero at 132 Å. This indicates that HCCNT3 can maintain its resistance to pullout for a longer duration than CCNT3 during the pullout process, despite having a lower maximum pullout force. The pullout energy curves for CCNTs and HCCNTs are presented in Figure 3d–f. Before the pullout of asphalt from the asphalt matrix, the energy values of CCNT1 and CCNT2 are generally consistent with those of HCCNT1 and HCCNT2. However, at the end of the pullout process, the pullout energy of CCNT1 and CCNT2 is relatively higher than that of HCCNT1 and HCCNT2, indicating that CCNT1 and CCNT2 exhibit greater pullout resistance in asphalt compared to HCCNT1 and HCCNT2. Furthermore, the trend of the pullout energy curves for CCNT3 and HCCNT3 differs from that of HCCNT1 and HCCNT2. The pullout energy of CCNT3 is much higher than that of HCCNT3 before 80 Å, but it drops sharply at distances beyond 90 Å, reflecting a sudden decrease in the pullout force of CCNT3, which corroborates the previous curves. Interestingly, the pullout energy of HCCNT3 surpasses that of CCNT3 at a distance of 130 Å, and their values become nearly the same at 160 Å. This suggests that HCCNT3 ultimately demonstrates superior performance in sustaining pullout energy compared to CCNT3 at longer pullout distances, highlighting the enhanced stability of HCCNT3.

**Figure 4:**
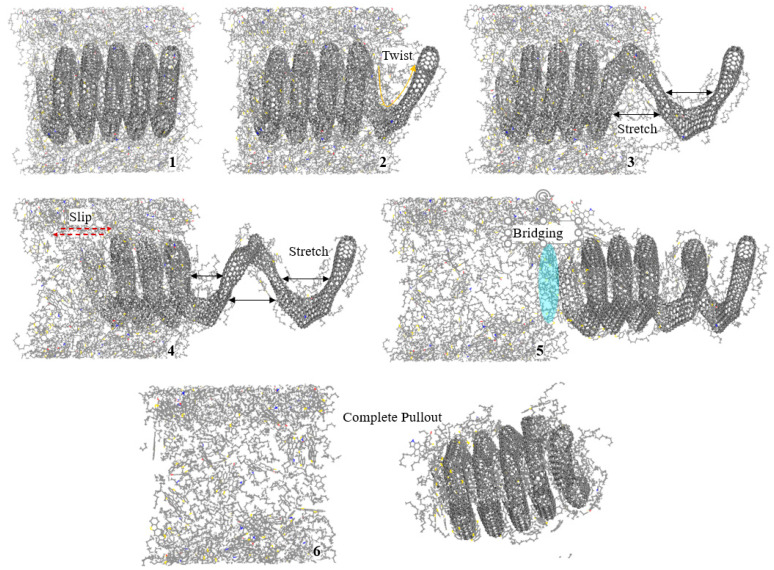
Snapshots of CCNT3 pulled out from the asphalt matrix. The molecular structure of CCNT3 undergoes twisting, stretching, bridging, and finally detaching from the asphalt matrix.

The molecular details of CCNT3 and HCCCNT3 extracted from the asphalt matrix are analyzed to investigate the distinct behaviors that occur during their pullout process. The snapshots of CCNT3 being pulled out from the asphalt matrix are shown in Figure 4. It is observed that the entire CCNT3 structure remains embedded in the asphalt matrix before the pullout, while the front loop of CCNT3 twists when the pullout force is applied. As the pullout force increases, noticeable stretching occurs between the first and second loops of CCNT3 along the pullout direction. However, the entire CCNT3 body does not exhibit significant relative movement with the asphalt matrix. In the fourth snapshot, a clear slip is observed between CCNT3 and the asphalt matrix, and the second loop of CCNT3 begins to stretch at this moment, while the other loops do not stretch. This indicates that the maximum pullout force is reached just before the slipping occurs. The response of these structures is non-uniform when an external force is imposed to stretch them. In the fifth snapshot, CCNT3 is on the verge of being fully pulled out from the asphalt matrix, and it can be noted that the front loops of CCNT3 contract slightly as less of it remains embedded in the asphalt matrix, while still maintaining a connection to it. The sixth snapshot shows the complete pullout of CCNT3 from the asphalt matrix, during which a significant amount of asphalt molecules is dragged out as well. At this point, the morphology of CCNT3 returns to its original shape, and all stretching has disappeared. This suggests that an abrupt drop in pullout force occurs at the moment CCNT3 is released from the matrix, as the spring structure of CCNT3 behaves like a spring, contracting back and exerting a reverse force.

**Figure 5:**
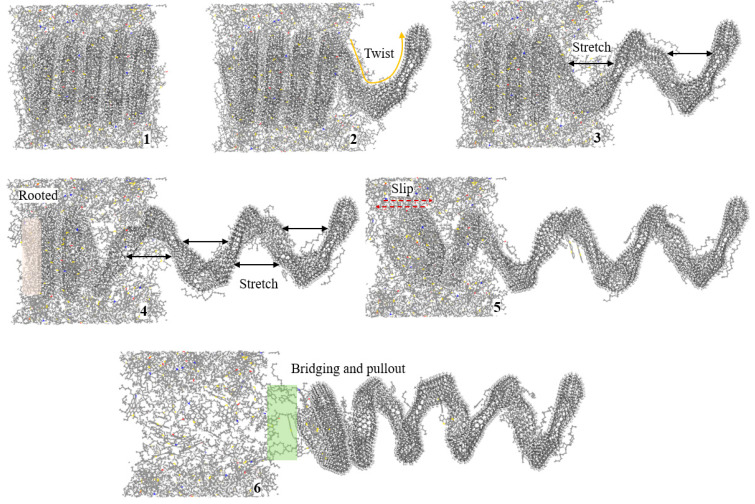
Snapshots of HCCNT3 pulled out from the asphalt matrix. The front three loops of HCCNT3 are fully stretched prior to the complete pullout of HCCNT3.

The snapshots of HCCNT3 pulled out from the asphalt matrix are presented in Figure 5. Similarly to CCNT3, the main body of HCCNT3 is fully merged in the asphalt matrix, and the front loops of HCCNT3 are twisted and stretched at the initial stage of the pullout, while the rest remains inside the matrix. Unlike CCNT3, in the fourth snapshot, the third loop of HCCNT3 is fully stretched, yet there is still no relative slip, and the rest of HCCNT3 remains rooted in the asphalt matrix. This finding corresponds to the sustainable pullout force of HCCNT3 when the pullout distance exceeds 80 Å. It is suggested that the pullout flexibility of HCCNT3 is higher than that of CCNT3, which allows for more stretching during the pullout process. As observed in the fifth snapshot, slipping between HCCNT3 and the asphalt matrix occurs when all four front loops are fully stretched, which is reflected by the deflection of the rooted loops in the fourth snapshot. At the same time, asphalt molecules can attach to the internal sections of the loops and be pulled out from the matrix. As shown in the sixth snapshot, the stretching extent of HCCNT3 decreases as the main body is about to move out of the asphalt matrix. Some asphalt molecules, especially saturate molecules with chain structures, are bridging between the asphalt matrix and HCCNT3.

Compared to CCNT3, a higher extent of deformation of HCCNT3 can be observed during the pullout process, indicating the greater flexibility of HCCNT3. This finding also aligns with previous results that show HCCNT3 exhibits greater flexibility and stretchability during uniaxial tensile testing [19]. This discovery is crucial because asphalt is a viscoelastic material, and HCCNT3 exhibits greater flexibility and stretchability, which is critical for modified asphalt as it enhances the material’s ability to withstand deformation under stress and temperature variations. This flexibility allows the asphalt to better absorb and dissipate energy, reducing the risk of cracking and improving overall durability.

The mean-squared displacement (MSD) can be determined to demonstrate the transitional mobility of the center of mass of asphalt molecules throughout the pullout process. It is defined as

(2) $$MSD(t)=\langle \Delta r_{i}{\left(t\right)}^{2}\rangle =\langle {\left(r_{i}(t)-r_{i}(0)\right)}^{2}\rangle$$

where *r_i_*(*t*) denotes the position vector of particle *i* at time *t*, and the angular brackets represent the average distance traveled by the particles.

**Figure 6:**
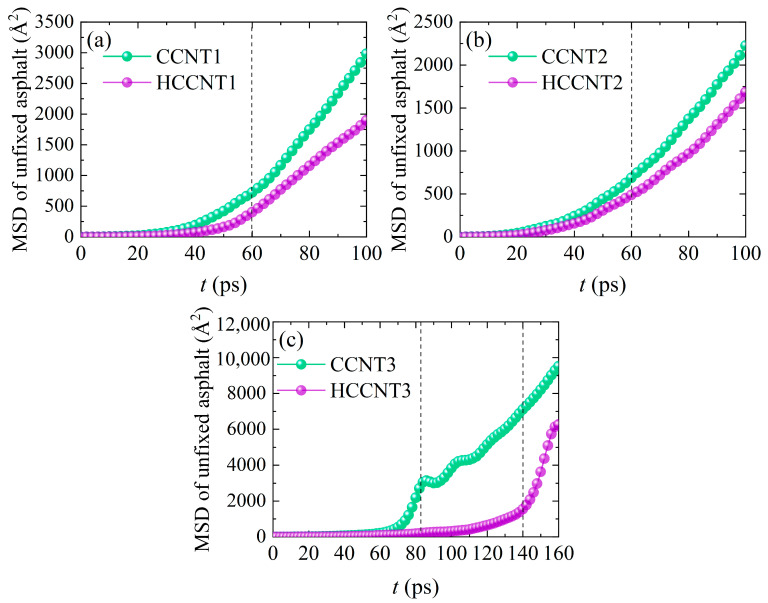
MSD curves of unfixed asphalt matrix during the pullout process for (**a**) CCNT1 and HCCNT1, (**b**) CCNT2 and HCCNT2, (**c**) CCNT3 and HCCNT3 (The vertical dash lines in (**a**,**b**) refer to the moments at which CCNT1/HCCNT1 and CCNT2/HCCNT2 are completely pulled out from the asphalt matrix. The first vertical dash line and the second vertical dash line in (**c**) refer to CCNT3 and HCCNT3 pulled out from the asphalt matrix, respectively).

To quantitatively evaluate the asphalt molecules pulled out by CCNTs and HCCNTs from the matrix, the MSD curves of the unfixed asphalt matrix for different CCNTs and HCCNTs are presented in Figure 6. The MSD was calculated for the asphalt molecules within the entire unfixed asphalt matrix. As shown in Figure 6a,b, the MSD values increase slowly up to 60 ps, indicating the partial pullout stage where the nanostructures are only partially embedded in the asphalt matrix. During this stage, the movement of asphalt molecules occurs primarily in the sections of the front loops of CCNTs and HCCNTs, resulting in relatively limited displacements of the unfixed asphalt molecules. At a key pullout moment of 60 ps, the MSD value for the unfixed asphalt matrix surrounding CCNT1 is 725 Å^2^, which is 1.8 times greater than the 403 Å^2^ observed for HCCNT1. Similarly, the MSD value for CCNT2 is 1.4 times higher than that of HCCNT2 at the same time point. This indicates that significantly more asphalt molecules can be extracted from the matrix by the CCNTs compared to their hydrogenated counterparts. For CCNT3 and HCCNT3, the MSD curves remain nearly identical up to 70 ps, but they diverge beyond that distance. Notably, there is an inflection point at approximately 80 ps in the MSD curve for CCNT3, signifying a sudden decrease in the displacement values of the unfixed asphalt molecules as CCNT3 is fully pulled out of the matrix, leaving some asphalt molecules behind instead of being dragged onto its surface. In contrast, the inflection point for HCCNT3 occurs around 140 ps, with the MSD values for HCCNT3 increasing rapidly beyond this distance. At this point, the MSD value for the unfixed asphalt matrix surrounding CCNT3 is 4.6 times greater than that of HCCNT3. Overall, the MSD values of CCNT3 are higher than those of HCCNT3 throughout the pullout process, indicating that more asphalt molecules are influenced by CCNT3. This can be attributed to the differences in carbon bond nature between the sp^2^ bonds of CCNTs and the sp^3^ bonds of HCCNTs [49]. The stronger π-π stacking interactions between CCNT3 and asphalt molecules facilitate the movement of asphalt molecules along the pullout direction.

**Figure 7:**
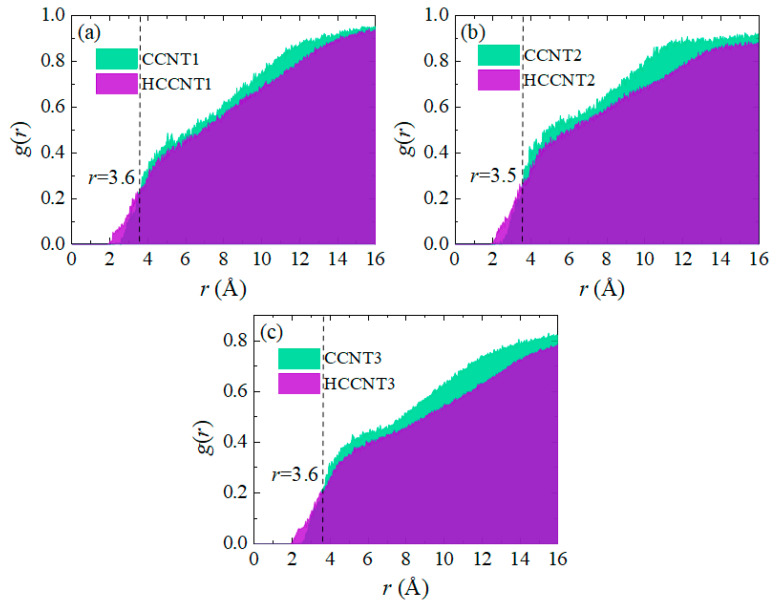
RDF curves between (**a**) CCNT1/HCCNT1, (**b**) CCNT2/HCCNT2, and (**c**) CCNT3/HCCNT3 and asphalt molecules and their integration evolution (The colored areas below the curves refer to the integration of the *g*(*r*) values).

The radial distribution function (RDF), represented as *g*(*r*), is utilized to quantitatively analyze the spatial configuration between CCNTs/HCNTs and asphalt molecules, as illustrated in Figure 7. The *g*(*r*) value is defined as the ratio of the average local number density of particles at a distance *r* to the bulk particle density *ρ*, expressed mathematically as follows:

(3) g(r)=〈ρ(r)〉/ρ

The analysis reveals that the *g*(*r*) values for both CCNTs and HCCNTs are zero within 2 Å. For HCCNTs, the *g*(*r*) values begin to rise at 2 Å, whereas for CCNTs, this increase occurs around 2.5 Å. This distinction can be attributed to the surface chemistry of HCCNTs, particularly the presence of hydrogen atoms, which enhance interactions with asphalt molecules at shorter distances compared to CCNTs. Consequently, HCCNTs exhibit a more pronounced increase in local density at these closer proximities. As the distance increases, the *g*(*r*) values for CCNTs surpass those of HCCNTs, reaching 3.6 Å for CCNT1, 3.5 Å for CCNT2, and 3.6 Å for CCNT3. This observation is consistent with previous studies, which report that *g*(*r*) values tend to increase rapidly starting from 3.5 Å [33], corresponding to the typical interplanar distance associated with π-π stacking, which ranges between 3.5 Å and 4 Å. Moreover, it is noteworthy that the integrated areas of *g*(*r*) values for CCNTs are relatively higher than those for HCCNTs. This indicates a greater average local density of asphalt molecules surrounding CCNTs, suggesting that CCNTs have a stronger attraction for asphalt molecules within a specific distance. This finding aligns with our earlier results, which demonstrated that CCNTs can extract more unfixed asphalt molecules from the matrix compared to HCCNTs, highlighting the enhanced interaction between CCNTs and asphalt.

**Figure 8:**
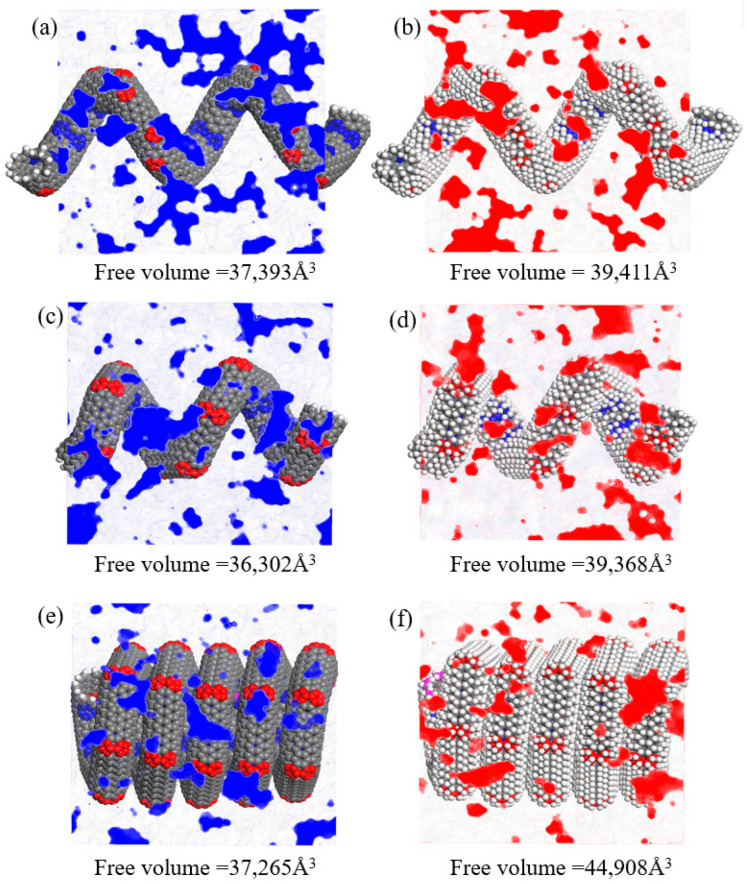
Free volume distribution and values in (**a**) CCNT1-, (**b**) HCCNT1-, (**c**) CCNT2-, (**d**) HCCNT2-, (**e**) CCNT3-, and (**f**) HCCNT3-modified asphalt nanocomposites at 300 K.

The free volume within asphalt nanocomposites can develop into small cavities when subjected to external loads. The propagation of these cavities can connect and lead to the formation of nano-cracks. Moreover, the pullout performance of asphalt nanocomposites modified with CCNTs and HCCNTs can be significantly affected by the enlargement of free volume. The larger free volumes can lead to lower cohesive energy density, as the additional space allows for increased molecular mobility and reduced intermolecular interactions. This reduction in cohesive energy density subsequently results in lower pullout energy, since less energy is required to detach molecular entities from the material. Consequently, the Connolly surface method [50] is employed to characterize the free volume in these asphalt nanocomposites. The Connolly probe radius in calculating the atom volume field is specified as 1.0 Å, while the grid resolution is set to be 0.4 Å. The free volume values of the three replicas are averaged to obtain the final free volume value. The distribution and values of free volume for CCNTs and HCCNTs modified asphalt nanocomposites at 300 K, after achieving full equilibrium, are presented in Figure 8. The free volume values of CCNT1, CCNT2, and CCNT3 are 37,393 Å^3^, 36302 Å^3^, 37,265 Å^3^, respectively, while the free volume values of HCCNT1, HCCNT2, and HCCNT3 are 39,411 Å^3^, 39,368 Å^3^, 44,908 Å^3^, respectively. It is evident that the free volumes of HCCNTs modified asphalt nanocomposites are larger than those of CCNTs modified asphalt nanocomposites. This suggests that the hydrogenation of CCNTs results in a greater capacity for developing nano-damage under external loads, which can lead to reduced pullout performance. Previous studies indicate that hydrogenation introduces C-H bonds that convert certain sp^2^ carbons into sp^3^-like configurations, relaxing π-bond networks and generating point defects. This process can decrease local stiffness and alter failure pathways under load, thereby increasing the likelihood of nano-damage initiation at these defect sites [19]. Additionally, CCNT2 modified asphalt nanocomposite has the smallest free volume, while HCCNT3 modified asphalt nanocomposite exhibits the largest free volume. The increase in free volume at the HCCNT3/asphalt interface results in the debonding of HCCNT3, which facilitates the free movement of asphalt molecules around the DNT surface. Due to its superior flexibility and stretchability, HCCNT3 can move more freely along the pullout direction because of its greater free volume.

**Figure 9:**
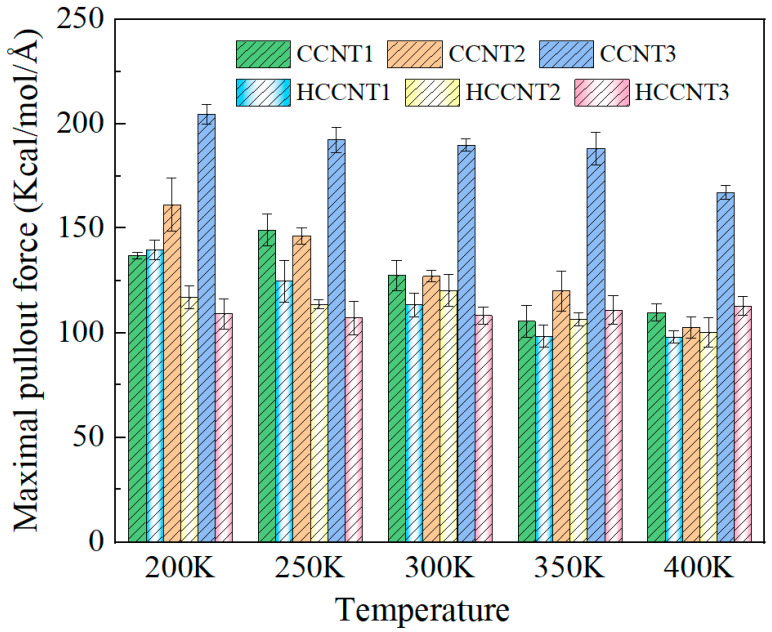
Maximum pullout force of CCNT/HCCNT-modified asphalt nanocomposites at different temperature ranges.

As a temperature-sensitive material, temperature variations can significantly affect the mechanical properties and performance of asphalt nanocomposites [51]. The maximum pullout force values of CCNTs and HCCNTs modified asphalt nanocomposites at different temperature ranges have been calculated and are presented in Figure 9. The selected temperature range of 200–400 K encompasses the typical service temperatures of asphalt pavements from approximately −30 °C to 80 °C and the extreme conditions associated with freeze–thaw cycles and diurnal temperature variations. It can be observed that the maximum pullout force of nearly all CCNTs/HCCNTs modified asphalt nanocomposites shows a decreasing trend as the temperature increases from 200 K to 400 K, suggesting that temperature plays a detrimental role in the pullout properties of these nanocomposites. It has been proven that the increase in temperature leads to a reduction in the bond strength between the carbon nanotubes and the asphalt matrix, resulting in decreased pullout force [48,52]. Among the various CCNTs/HCCNTs, CCNT3 exhibits the highest maximum pullout force, which decreases from 204 kcal/mol/Å at 200 K to 167 kcal/mol/Å at 400 K, representing a 20% reduction. In contrast, the maximum pullout force of HCCNT3 remains relatively stable at around 110 kcal/mol/Å, indicating that its pullout performance is less susceptible to temperature variations compared to CCNT3. Additionally, except for CCNT1 and HCCNT1 at 200 K, it is noted that the maximum pullout forces of CCNTs are generally higher than those of HCCNTs across all tested temperature ranges, indicating that CCNTs modified asphalt nanocomposites achieve better pullout performance compared to HCCNTs. At 200 K, the maximum pullout force of CCNT1 is approximately 1.5 times greater than that of HCCNT3; however, the difference between these two decreases from 200 K to 350 K, and ultimately, at higher temperatures, the maximum pullout force of CCNT1 falls below that of HCCNT3. This indicates that the stability or strength of CCNT1 deteriorates under high-temperature conditions, leading to its pullout performance being inferior to that of HCCNT3. It also suggests that the HCCNT3 exhibits greater thermal stability primarily due to the presence of C-H bonds, which help to mitigate the sensitivity of π-π stackings of CCNTs to temperature fluctuations. In conclusion, CCNTs modified asphalt nanocomposites demonstrate superior pullout performance compared to HCCNTs, while the pullout performance of HCCNTs modified asphalt nanocomposites is more stable under temperature variations.

**Figure 10:**
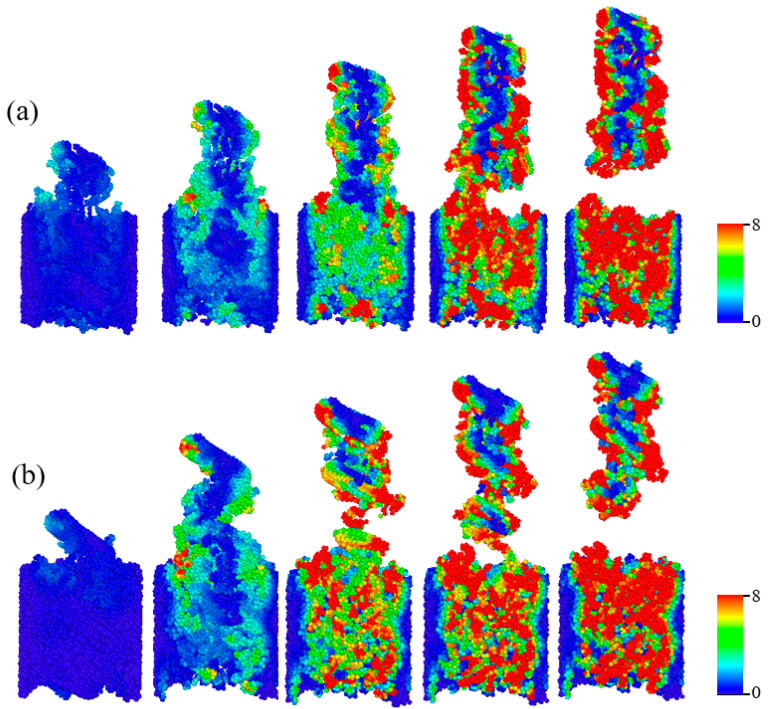
Snapshots of the local atomic deformation gradients of (**a**) CCNT1- and (**b**) HCCNT1-modified asphalt composites during the pullout process.

To evaluate the atomistic deformation of asphalt nanocomposites during the pullout process, the local atomic deformation gradients of CCNT1/HCCNT1-modified asphalt nanocomposites are presented in Figure 10. It shows that at the beginning of the pullout, the local atomic deformations occur at the front loops of CCNT1/HCCNT1, which is the pullout point, as well as in the asphalt matrix close to this point. As the pullout progresses, the deformation extends deeper into the asphalt matrix, generating two apparent interfaces between the asphalt matrix and CCNT1/HCCNT1. This indicates a relative slippage between them. However, the local atomic deformation within the CCNT1/HCCNT1 structures remains small. As shown in the snapshots of the second row, the local atomic deformation of HCCNT1 is noticeably greater than that of CCNT1, indicating the higher flexibility and stretchability of HCCNT1. When the nanostructures are about to be extracted from the matrix, the local atomic deformation of asphalt molecules between CCNT1/HCCNT1 and the asphalt matrix increases rapidly, highlighting the bridging effect of these molecules. Once the nanostructures are completely pulled out from the asphalt matrix, it is clearly observed that CCNT1 carries more asphalt molecules, resulting in a higher local atomic deformation compared to HCCNT1. Additionally, both CCNT1 and HCCNT1 nanostructures return to their original shapes with similar local atomic deformations. It is found that the local atomic deformations of the asphalt matrix are more concentrated in the central sections throughout the entire pullout process, beginning at the interface area and evolving toward the central area. The local atomic deformations of CCNT1/HCCNT1 are larger at the edge regions and decrease toward the central axis.

## 4. Conclusions

This study investigates the pullout performance of CCNTs and HCCNTs in reinforcing asphalt nanocomposites, analyzing their reinforcing mechanisms through MD simulations. The results indicate that CCNTs generally exhibit superior maximal pullout forces compared to HCCNTs, particularly at lower temperatures, which suggests stronger adhesion within the asphalt matrix. In contrast, HCCNTs exhibit improved stability and flexibility under varying temperature conditions, allowing them to maintain more pullout energy over longer distances during the pullout process. Analyses of molecular movement reveal that CCNTs facilitate greater displacement of asphalt molecules, indicating stronger interactions, while spatial configuration analyses show that CCNTs attract more asphalt molecules in close proximity, thereby enhancing their effectiveness within the composite. Furthermore, the examination of free volume distribution highlights that HCCNTs possess a larger free volume. This characteristic may increase the risk of nano-damage inside asphalt nanocomposites, but it also allows for greater flexibility during the pullout process. Overall, these findings highlight the essential role of CCNTs and HCCNTs in enhancing the pullout performance of asphalt materials. In addition, the distinct behaviors and contributions of these nanomaterials on modifying asphalt are elucidated, which ultimately leads to the development of more resilient and sustainable asphalt pavements.

The limitations of this study include the following: the simplified model may not adequately represent the extensive chemical diversity in asphalt, leading to an incomplete understanding of its properties and behaviors. Additionally, it does not account for interactions among a broader range of chemical species, which can significantly affect performance. While CVFF is a widely used force field, it may not fully capture the unique characteristics of graphitic structures, such as anisotropic properties and specific π-π interactions that are essential for accurate modeling.

The future directions of this study should focus on several critical areas to enhance the understanding and application of CCNTs and HCCNTs in asphalt technology: 1. Investigating the long-term durability and aging effects of these nanostructures in asphalt under real-world environmental conditions is essential, as this will ensure their reliable performance and contribute to the longevity of asphalt pavements. By assessing how these materials withstand various environmental factors over time, we can better predict their lifespan and maintenance needs. 2. Examining the impact of structural defects on the performance of CCNTs and HCCNTs within asphalt materials is crucial. Understanding how these defects influence mechanical properties will enable us to optimize their design and integration, thereby enhancing their effectiveness as reinforcements. This knowledge will help in tailoring the nanostructures to minimize weaknesses and maximize their reinforcing capabilities. 3. Exploring the synergistic effects of combining CCNTs or HCCNTs with other asphalt modifiers can lead to innovative asphalt formulations. By developing formulations that effectively integrate these nanostructures with other materials, researchers can create asphalt that better withstands environmental stresses and mechanical loads, ultimately resulting in longer-lasting and more resilient road surfaces. 4. Exploring the use of reactive force fields in CCNTs/HCCNTs modified asphalt nanocomposites, which could provide a more accurate representation of the chemical reactions and interactions within asphalt systems. 5. Developing larger and more comprehensive asphalt models, which would allow for a deeper understanding of the asphalt material’s behaviors under various environmental conditions. 6. Incorporating experimental validation of our computational results, which can strengthen the reliability of our findings and bridge the gap between theoretical predictions and practical applications. By addressing these areas, future research can significantly advance the potential applications of CCNTs and HCCNTs in asphalt technology, paving the way for the development of more durable asphalt nanocomposites.

## Figures and Tables

**Table 1:** Mass ratios of the asphalt components.

Asphalt Model	Mass (g/mol)	Chemical Formula	Number of Molecules	Total Mass (g/mol)	Mass Fraction (%)
Asphaltene	754.0	C_53_H_55_NOS	14	10,556.0	25.6
Polar aromatic	290.4	C_18_H_10_S_2_	24	6969.6	16.9
Naphthene aromatic	156.2	C_12_H_12_	21	3280.2	7.9
Saturate	310.6	C_22_H_46_	66	20,499.6	49.6
Asphalt binder			125	41,305.4	

## Data Availability

Data are available from the corresponding author upon reasonable request.
